# Characteristics and outcomes of therapy-related myeloid neoplasms following autologous stem cell transplantation for multiple myeloma

**DOI:** 10.1038/s41408-021-00454-y

**Published:** 2021-03-19

**Authors:** Kalyan Nadiminti, M. Hasib Sidiqi, Kapil Meleveedu, Hassan B. Alkhateeb, William J. Hogan, Mark Litzow, Mrinal Patnaik, Shaji Kumar, Morie Gertz, Dong Chen, Mithun Vinod Shah

**Affiliations:** 1 Division of Hematology, Mayo Clinic, Rochester, MN USA; 2 Division of Hematology, Oncology, and Palliative Care, University of Wisconsin, Madison, WI USA; 3 Division of Division of Bone Marrow Transplantation, Roger Williams Medical Center, Providence, RI USA; 4 Division of Hematopathology, Mayo Clinic, Rochester, MN USA

**Keywords:** Myeloma, Risk factors

**Dear Editor**,

Advances over the last two decades have resulted in a significant improvement in survival of patients with multiple myeloma (MM)—primarily driven by the emergence of immunomodulators (IMiDs), proteasome inhibitors (PI) and monoclonal antibodies. Despite these improvements, high-dose melphalan (HDM)-based autologous stem cell transplantation (SCT) followed by lenalidomide maintenance remains part of standard initial therapy for transplant-eligible patients^1^. The development of therapy-related myeloid neoplasms (t-MN) is an uncommon, but dreadful complication with considerable morbidity and poor survival. While the exposure to melphalan and autologous SCT are known risk factors^2,3^, the impact of lenalidomide, in the setting of HDM, is less clear^4–7^. Autologous SCT followed by lenalidomide maintenance leads to an improved progression-free- and overall survival, making it the de facto standard for eligible patients^5,6^. We studied the incidence of t-MN in post-SCT MM patients in the era of novel therapies and studied the impact of lenalidomide exposure on the risk of t-MN post-SCT. We also analyzed factors that may impact outcomes after t-MN diagnosis.

We identified MM patients that underwent autologous SCT following HDM (1998–2016) and subsequently developed t-MN as defined by the 2016 World Health Organization classification. Complex and monosomal karyotype (CK and MK respectively) were defined according to the 1995 International System for Human Cytogenetic Nomenclature recommendations. Myeloid mutational analysis was obtained using the clinically available 43-gene next-generation sequencing (NGS) panel available at Mayo Clinic, Rochester. Overall survival (OS) was calculated using the standard Kaplan–Meier technique. Univariate and multivariate analyses using Cox proportional hazard model were performed to study the interaction of risk factors with the outcomes. Statistical analyses were performed using SAS (JMP v14.1) and GraphPad Prism (v7). Factors purported to predispose to t-MN development such as alkylator therapy including HDM, a second autologous SCT, and exposure to lenalidomide were analyzed. Outcome variables were assessed from the time of t-MN diagnosis, except for the cumulative incidence of t-MN, which was assessed from the time of MM diagnosis.

We found 2448 consecutive patients that underwent first autologous SCT for MM. Of these, 52 patients (2.1%) developed t-MN. Thirty-seven of 52 (71%) patients with t-MN had received lenalidomide. In contrast, of 2396 patients that did not develop t-MN, 1285 (54%) had received lenalidomide. Thus, lenalidomide exposure was associated a significantly higher risk of t-MN (*χ*^2^ with Yate’s correction 5.6, *P* = 0.002, Supplementary Fig. 1). Our analysis is in agreement with a recent report of t-MN in post-SCT patients that post-SCT lenalidomide was associated with a higher risk of t-MN and all patients that developed t-MN had been on lenalidomide or thalidomide^7^. To account for lenalidomide-associated survival bias, a time-dependent analysis of the incidence of t-MN was performed. There was a significant increase in the incidence of t-MN among the patients that received lenalidomide compared to those who did not (*P* < 0.0001, Supplementary Fig. 2). Patients that developed t-MN had a higher median number of apheresis sessions compared to those that did not develop t-MN (3 vs. 2, *P* = 0.0007, Supplementary Fig. 3), while the number of median CD34^+^ cells collected did not differ (8.8 vs. 9.2, *P* = 0.5). Exposure to alkylators [melphalan, cyclophosphamide, low-dose melphalan, BCNU] and anthracycline (AC, yes vs. no) did not predict the development of t-MN (data not shown).

Nine patients were excluded due to lack of follow-up or unavailability of clinical data. Baseline characteristics of 43 patients shown in Table 1. Thirty-one (72%) were males and the median age at diagnosis was 70 years (range 44–79) for t-MDS and 61 years (range 51–77) for t-AML. Median time from autologous SCT to t-MN development for the entire cohort was 5 years (range 1–15) while it was 5 years (range 1–15) for MDS and 4 years (range 1–9) for AML. Seven patients (16%) presented with t-AML, and 36 (84%) with t-MDS. Pathologic and treatment features are tabulated (Supplementary Table 1). Of the 36 patients with t-MDS, 13 (36%) transformed to AML during their clinical course with the median time to transformation being 6 months (range 3–17). MK and CK were predominant, accounting for 51% each in t-MDS, and 57% and 72% of patients with t-AML, respectively. NGS at the time of t-MN diagnosis was available for 13 (30%) patients, of which 7 (50%) had mutated *TP53*.

**Table 1:** Clinical and pathologic characteristics of multiple myeloma (MM) patients that developed therapy-related myeloid neoplasm (t-MN).

Characteristics	t-MDS (*n* = 36)	t-AML (*n* = 7)
Age at MM diagnosis (years), median (range)	61 (41–74)	58 (47–73)
Age at autologous SCT (years), median (range)	64.5 (41–74)	59 (48–73)
Age at t-MN diagnosis (years), median (range)	70 (44–79)	61 (51–77)
Sex, male (%)	25 (70%)	6 (85%)
Time from autologous SCT to t-MN (years), median (range)	5 (1–15)	4 (1–9)
Transformed to AML, (%)	13 (36%)	n/a
Time to transform to AML (months), median (range)	6 (3–17)	n/a
Patients that underwent second SCT for MM, *n* (%)	6 (16%)	1 (14%)
Patients that received lenalidomide pre-SCT, *n* (%)	9 (21%)	3 (43%)
Patients that received lenalidomide post-SCT, *n* (%)	28 (66%)	6 (85%)
Patients that received lenalidomide ≥2 years post-SCT, *n* (%)	12 (33%)	4 (57%)
Cycles of chemotherapy containing alkylators, including HDM and cyclophosphamide for mobilization, median (range)	1 (0–6)	1 (1–4)
Proportion with blast % ≥10, *n* (%)	6 (17%)	n/a
Complex karyotype, *n* (%)	18 (50%)	5 (72%)
Monosomal karyotype, *n* (%)	25 (70%)	4 (57%)
Complex and monosomal karyotype, *n* (%)	13 (36%)	4 (57%)
Received hypomethylating agents for t-MN, *n* (%)	13 (38%)	1 (16%)
Allogeneic SCT for t-MN, *n* (%)	3 (8%)	1 (14%)
Autologous SCT for t-MN, *n* (%)	4 (11%)	None
Overall survival in months since t-MN, median (95% CI)	15 (10–18)	6 (2–14)

*t-MDS* therapy-related myelodysplastic syndrome, *t-AML* therapy-related acute myeloid leukemia, *MM* multiple myeloma, *SCT* stem cell transplant, *HDM* high dose melphalan, *t-MN* therapy-related myeloid neoplasm.

Initial treatment of t-MN included hypomethylating agents (HMA), lenalidomide, induction chemotherapy, or supportive care alone. Fourteen (32.5%) patients received only supportive care either due to poor performance status or due to a low-risk disease. Of the remaining 29 (67.5%), 13 (36%) and 1 (14%) received HMA as the frontline therapy among t-MDS and t-AML groups, respectively. Five (71.5%) patients with t-AML and 3 (8%) with t-MDS received induction chemotherapy and 6 (75%) required salvage therapy. Overall, 8 (19%) patients proceeded to receive an autologous SCT from previously collected PBSC (*n* = 4) or allogeneic (*n* = 4) transplant, of which only one patient was from t-AML group (Supplementary Table 2). The rest (21, 48%) did not proceed to SCT due to inadequate control of the disease.

After a median follow-up of 70 months [95% confidence interval (CI), 38–134], the median OS was 12 months (95% CI, 9–17, Fig. 1). There was no difference in survival for those with t-MDS or t-AML (15 vs. 6 months, *P* = 0.2), those who underwent the transplant, compared to those who did not (17 vs. 10 months, *P* = 0.3), or those who received allo SCT compared to autologous SCT (21 vs. 16 months, *P* = 0.7). At the time of the last follow-up, 9 (21%) patients were alive. Primary causes of death were progressive t-MN (71%), MM (12%), or both (6%). Among the eight patients that underwent any SCT, one person was alive at the last follow-up. Five (62.5%) patients died of t-MN, one each died of progressive MM, and transplanted-related mortality (Supplementary Table 2).

**Fig. 1:**
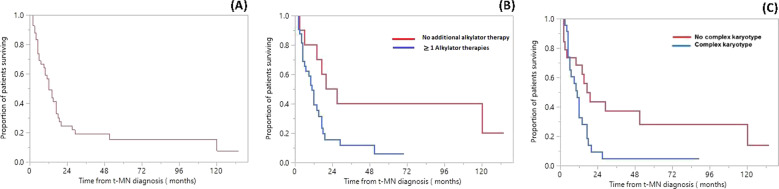
Survival of multiple myeloma (MM) patients that underwent autologous SCT and developed therapy-related myeloid neoplasm (t-MN). **A** Overall survival following t-MN diagnosis; **B** Overall survival following t-MN diagnosis for patients that received ≥1 cycle of alkylator therapy compared to those who received no additional alkylator therapy; **C** Overall survival following t-MN diagnosis for patients with a complex karyotype (CK) compared to those without CK.

We then ascertained the association of cumulative exposure to alkylator therapy with t-MN outcomes. Median number of alkylator/AC therapy cycles used prior to autologous SCT was 1 (range 0–6) among both t-MDS and t-AML groups. Seven (17%) patients received two autologous SCTs. On univariable analysis, factors that predicted OS following t-MN diagnosis were exposure to ≥1 line of alkylator therapies 11 vs. 27 months, (*P* = 0.02), the presence of ≥10% vs. < 10% blast at the time of t-MN diagnosis (5.5 vs. 17 months, *P* = 0.01), and the presence of CK vs. not (11 vs. 17 months, *P* = 0.03). There was no difference in survival among t-MDS patients who transformed to t-AML vs. those who did not, those who received 1 vs. 2 autologous SCT, and those with lenalidomide exposure duration ≥2 vs. <2 years. On multivariable analysis, the number of lines of alkylator therapies, the presence of CK, and the total % blasts remained as significant predictors of OS (Supplementary Table 3).

A unique aspect of our study is the availability of patient-specific disease and treatment characteristics that provide a better insight to the natural history of t-MN. In a large cohort of patients that received HDM and autologous SCT for MM, we observed a 5.6-fold increase in the risk of t-MN in patients receiving lenalidomide compared to those who did not, which is in agreement with a recent study^7^. Our results are consistent with the published experience of cumulative t-MN incidence ranging between 0.5–6% in the era of autologous SCT with HDM and extended use of novel agents, including IMiDs, in MM.

t-MN is a well-recognized complication occurring following exposure to cytotoxic chemotherapy and radiotherapy, including in those who receive autologous SCT. Complex karyotype and *TP53* mutations are very common across t-MN and portend a poor response to therapy and dismal survival, as we observed^3,8^. We found t-MDS as a more common initial presentation, as reported previously^8,9^—with nearly one-third transforming to t-AML eventually. We observed phenotypes of acute erythroid leukemia in three and megakaryoblastic leukemia in one patient respectively, which are known to be frequently associated with CK, *TP53* mutation and a dismal survival^10^. At the same time, about 16% of our patients had stable disease without treatment, indicating the heterogeneity of this entity. In patients that required therapy, the response to conventional AML therapies was poor as most patients required salvage therapies. Ultimately, we noted only two long-term survivors, arbitrarily defined as those who survived ≥5 years—one each following autologous and allogeneic SCT. This is consistent with prior reports in t-MN showing marginal benefit of autologous SCT with around 20% long-term survival, owing to preponderance of CK and *TP53* mutations^9,11^.

Limitations of our study include those of it being a retrospective, an observational study spanning 2 decades. Since our cohort consisted of patients undergoing autologous SCT, we were unable to identify the impact of HDM on t-MN development. Cumulative exposure to alkylators, the duration of lenalidomide therapy, and radiation were not consistently available for patients that did not develop t-MN, precluding analyses of their impact on t-MN development. Finally, the unavailability of NGS data for a majority of patients limited our capacity to evaluate the role of genetic factors in transplant outcomes.

Nonetheless, our results emphasize the cumulative risk of alkylators along with lenalidomide in the development of t-MN^4^. Whether the development of t-MN is strictly due to exposures such as chemotherapy or radiation is controversial. Patients with monoclonal gammopathy of unclear significance as well as those with MM that have not received MM-directed therapy, are at a higher risk of developing myeloid neoplasms^12,13^. Phenotypic abnormalities reminiscent of myelodysplastic syndrome (MDS-PA), present in up to 11% of MM patients at diagnosis^14^; and clonal hematopoiesis (CH), present in 21% patients undergoing SCT^7^ have been studied as potential biomarkers for t-MN. However, neither MDS-PA nor CH are predictive of t-MN. As conventional therapies for t-MN remain ineffective, there is an urgent need to identify factors that predict the development of t-MN and survival thereafter–our study is a step in that direction.

## Supplementary information

Supplementary_Information
