# Supplementary material for: Characteristics and outcomes of therapy-related myeloid neoplasms following autologous stem cell transplantation for multiple myeloma
Source: Blood Cancer J. 2021 Mar 19;11(3):63. doi: 10.1038/s41408-021-00454-y (PMC7979889; doi:10.1038/s41408-021-00454-y)
Supplement: Supplementary file 1 — Supplementary_Information [file 41408_2021_454_MOESM1_ESM.pdf]

Supplementary Figure 1. Exposure to lenalidomide is associated with an increased risk of therapy-related myeloid neoplasm (t-MN) in multiple myeloma patients undergoing autologous stem cell transplant.

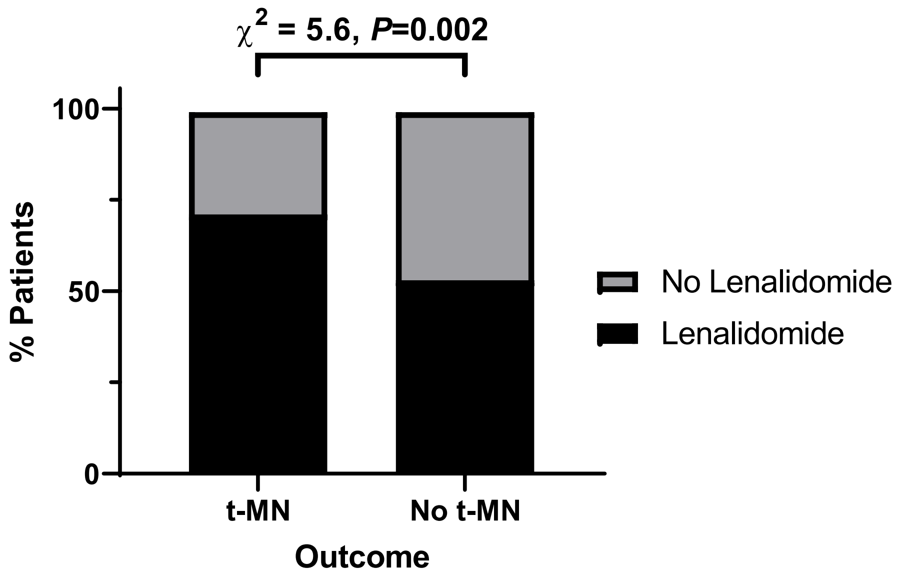

Supplementary Figure 2. Time-dependant analysis of incidence of therapy-related myeloid neoplasm (t-MN) among the lenalidomide who received lenalidomide therapy *vs*. those who did not

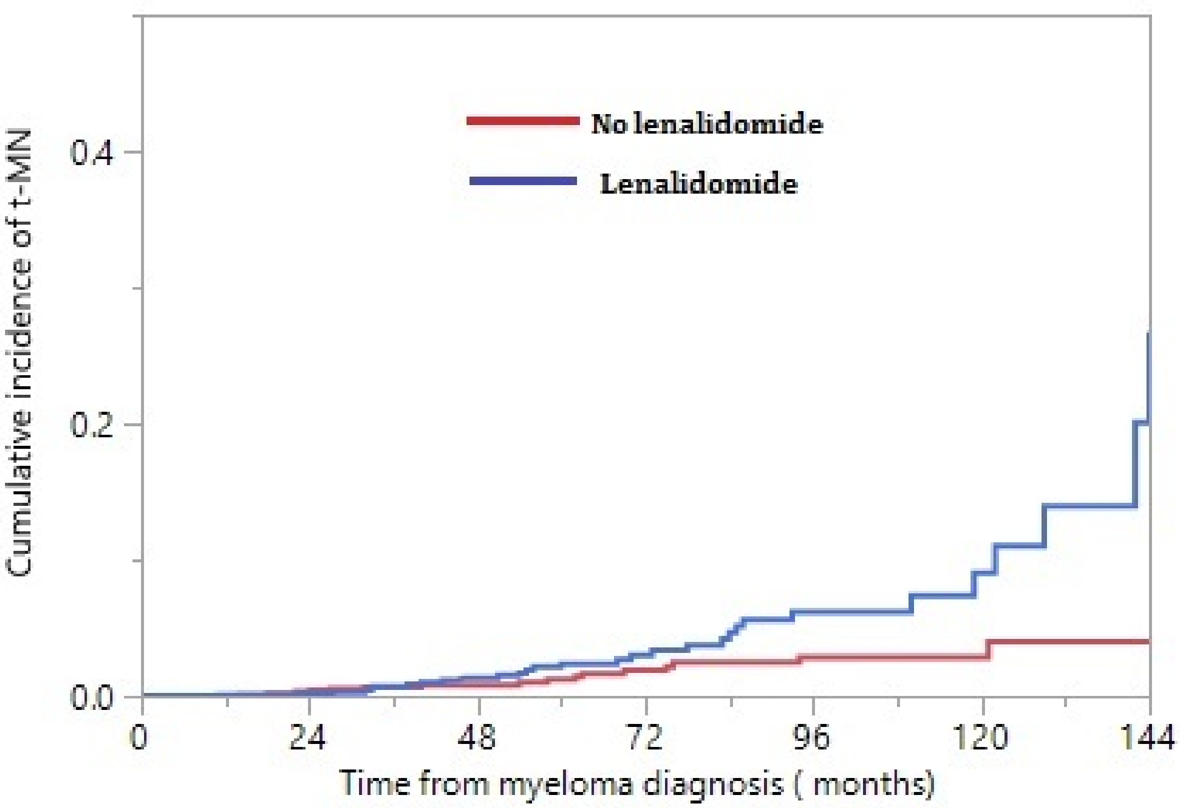

Supplementary Figure 3. Patients that developed therapy-related myeloid neoplasm (t-MN) underwent a higher number of apheresis compared to those who did not develop t-MN

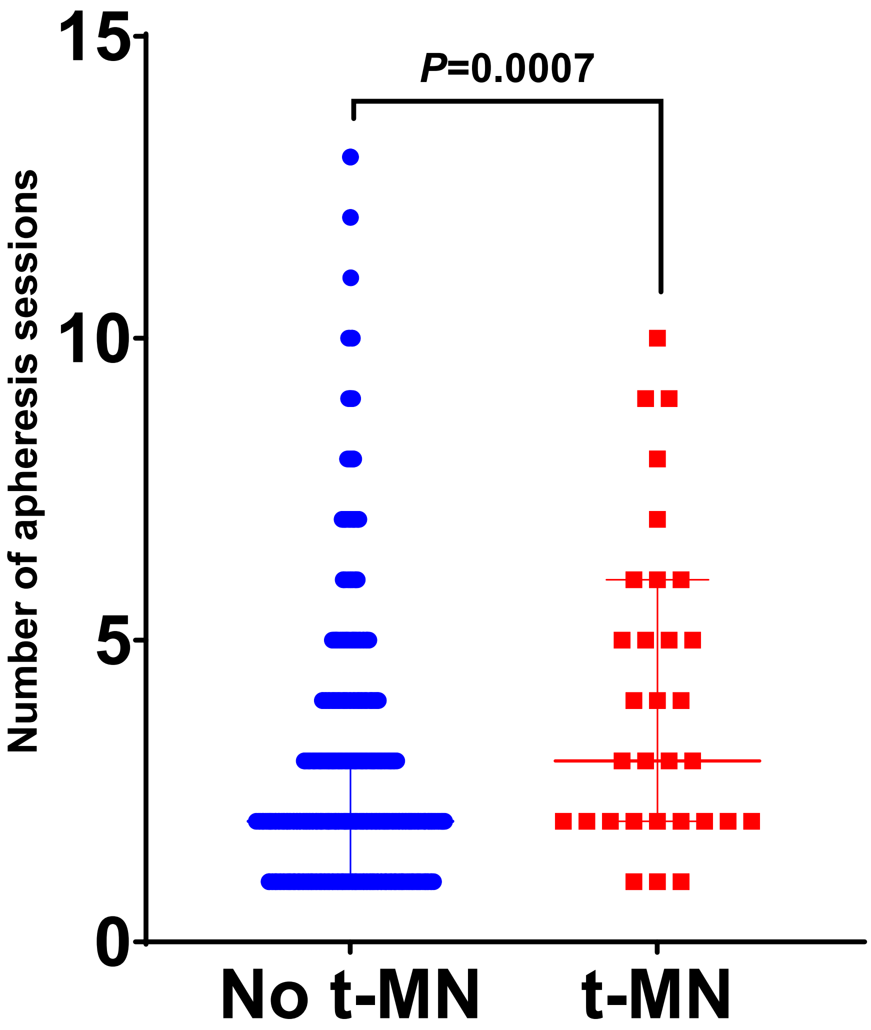

Supplementary Table 1. Clinical characteristics and treatment details of multiple myeloma (MM) patients that underwent autologous stem cell transplant (SCT) and developed therapy-related myeloid neoplasms (t-MN)

	t-MN (MDS/ AML)	Morphologic classification	Cytogenetics/FISH	NGS	BM blasts %	Treatment received?	Supportive Care	HMA	Treatment Details	Response
1	MDS	RAEB-1	45,XY,+der(1)t(1;15)(q12;q22),der(1;6)(q10;p10),add(5)(q31), t(6;17)(q10;p10),-8,add(13)(q14),add(14)(q24),-15,+18,-20, +mar[2]/45,X,-Y[5]/46,XY[13]	ND	8	Yes	Transfusions	Yes	decitabine	Progressed
2	MDS	RCMD	45,X,-Y,del(20)(q11.2q13.3)[17]	ND	5	No	None	no		Stable
3	MDS		-4,- 11, -16,-del (7q)	*DNMT3A*, *TP53* x 2	0	Yes	Transfusions	Yes	5-azacytidine x 9	Progressed
4	MDS	RAEB-2	47,XY,+8[1]/50-49,idem,+14,+21,+0-1mar[2]/47,XY,+r[cp2]/46,XY[15].	*ETV6,RUNX*1, *ZRSR2*	7	Yes	Transfusions	Yes	5-azacytidine x 4	Stable
5	AML	Pure erythroid leukemia	46,XY,der(17)t(15;17)(q15;p12)[3]/46-50,idem,add(4)(p15)[3], -7[4], add(11)(p11.1)[2], add(14)(p13)[5], -15[11], -16[10], add(18)(q21)[7], del(19)(p13)[9], -21[12],del(22)(q13)[5], +r[4], +mar1[11], +mar2[11], +mar3[3], +1-6mar[cp14]/46,XY[3].	ND	18	Yes	Transfusions	No	7+3(dauNo 90) x 2	Complete remission
6	MDS	RCMD	-5, -20, -7q,	ND	2	Yes	ESA, Lenalidomide	No		Progressed
7	MDS	Pure erythroid leukemia	45-49,+1,+8,+10,add(17)(p11.2),-19,-20,+22,+1-3mar[cp4]/45,XY,-5,add (17)(p11.2)[3]/46,XY[13].	*TP53*	2	Yes	Transfusions, ESA	No	lenalidomide x 3 months, Aranesp	Progressed
8	MDS	RAEB-1	- 5,- 7,- 20,- 21, + 8 and multiple structural abnormalities	*BCOR, TP53*	7	Yes	Transfusions	Yes	decitabine x 3	Stable
9	MDS	RCMD	del 5q, -7, del 3q, t (3;19)	ND	1	Yes	oral B6, ESA, Transfusions	No		Stable
10	MDS	RAEB-1	- 5,-7,-18, .	*TP53*	2	Yes	ESA,	Yes	decitabine x 3	Progressed

11	MDS		MK/ de (5q-) del 7q- del(8q24.1) MYC region , MLL amp (11q23)	ND	2	Yes	Transfusions	Yes	5-azacytidine	Partial remission
12	AML		CK	*TP53, and DNMT3*		Yes	Transfusions	Yes	CLAG-M, decitabine x 3, vaccine trial, Venetoclax+LDAC	Relapsed/ Refractory
13	MDS	RAEB-2	MK/ 54,XY,+Y,-5,der(7)t(1;7)(p22;q31),+8,+8,+9,+11,-13,+14,+15,+20,+2mar	*PDGFR beta mutation*	10	Yes	Transfusions	Yes	5-azacytidinex3, 7+3, MEC salvage,	Progressed
14	MDS		46,XY,del(5)(q13q33)[8]/46,idem,add(22)(p11.2)[3]/46,XY[9]	ND	0	No	Observation	No		Stable
15	MDS	RAEB-2	Diploid	ND	15	Yes	Transfusions	Yes	decitabine x 3	Stable
16	MDS	RAEB-2	44-50,XX,-3,add(5)(q11.2),del(7)(q11.2),-10,-12,add(15)(q22),+add(16)	ND	15	Yes	Transfusions	Yes	5-azacytidine x 2	Progressed
17	MDS		+ 21	*SRSF2, U2AF1*	3	Yes	Transfusions	No	CPX-351 induction	Progressed
18	MDS	RAEB-2	del (5q)	ND	10	Yes	None	No	Auto-SCT	Complete remission
19	MDS	NOC	t(1;10), del (20q)	ND	1	No	Transfusions	No		Progressed
20	MDS	MDS-MLD	Diploid	ND	0	No	None	No	Withheld lenalidomide	Remission
21	MDS	RCMD	46,XY, del (7q)+1,der(1;7)(q10;p10)[2]/46,XY[18].	ND	0	No	None	No	None	Complete remission
22	MDS	RCMD	del (20q)	*U2AF1*	2	No	None	No		Stable
23	MDS	RAEB-2	Diploid	ND	11	Yes	Transfusions	Yes	5-azacytidine x 3, 7+3	Progressed
24	MDS	RCMD	del(5q) - 7, del(17p), del (20q)	ND	3	Yes	Transfusions	Yes	relapse treated with decitabine	Progressed
25	AML	Monocytic	46,XY,t(9;11)(p22;q23	ND	90	Yes,	Transfusions, g-CSF, Hydrea	No	Ara-C, Hydrea	Progressed
26	MDS	RAEB-1	45,XY,-3,i(4)(q10),add(5)(q11.2)[6]/46,idem,+mar[2]/45,XY,-3,add(4)(p12),add(5)(q11.2)[5]/46,XY[7].	ND	7	Yes		No	Auto SCT	Progressed
27	MDS	RAEB-2	del(5q), del (3q21), del (7q)+8	ND	10	Yes	Transfusions	Yes	5-azacitidine, ErliNostat trial x 1 cycle	Progressed

28	MDS		46,XY,der(5;21)(p10;q10),-13,add(14)(p11.2),-20,+3r[19]/46,XY[1]	*TP53*	1	No	Observation	No		Stable
29	MDS	MDS/MPN-U	45,XX,-7[4]/45,idem,del(3)(q21)[1]/46,XX[15]	ND	1	Yes	Transfusions	Yes	decitabine x 16	Progressed
30	MDS	RAEB-2	+11	ND	15	No	Hospice	No		Progressed
31	MDS	RAEB-2	del (5q31), del(7q31), del (2q12) and + 8	ND	15	Yes	Transfusions, ESA	Yes	5-azacitidine x 3	Progressed
32	MDS		-7, del (17p), MLL	ND	2	No	G-CSF, Transfusions	No	None	Progressed
33	MDS		44,XX,add(5)(q11.2),der(7)t(7;17)(p13;q11.2),-17,-22[20]	ND	1	No	Transfusions, ESA	No		Progressed
34	AML	Megakaryo blastic	del (5q), 41-43,X,-Y,add(3)(q25),add(4)(q31.1),add(5)(q11.2), add(8)(q24.1),-9,+11, add(11)(q21),add(13)(p11.2),-15,-16,-19,-21,-22,+0-1r, +1-2mar[cp12]/46,XY[3]	ND	20	Yes		No	7+3(Ida), MEC	Complete remission
35	AML		t(11;12;21)	*RUNX1* .	60	No	Transfusions			Progressed
36	MDS	RAEB-2	45,XX,add(4)(q21),der(5)t(1;5)(p13;q15),add(7)(q11.2), -17[9]/46,sl,+9[4]/46,sl,+mar[3]/48-51,sl,-add(4)(q21), +add(4)(q12),add(9)(q34),5-8mar,+0-2r[cp4]	ND	50	Yes	Transfusions	No	7+3, 5+2, clofarabine	Progressed
37	MDS		46 XY at diagnosis evolved to CK at progression	*DNMT3A*	0	Yes	Transfusions, ESA,	No	Lenalidomide	Stable
38	MDS	RCMD	-5, - 7	ND	0	No	Transfusions	No	None	Progressed
39	MDS	RAEB-2	43-46,XX,-4,del(5)(q14q33),-17,add(21)(q22),-22,+1~4mar[cp10]/44,i , dem,add(14)(p10),-18[cp3]/43-45,idem,add(5)(p13),-18[cp2]/44-46,idem, del(7)(q22q34)[cp4]/43-45,idem,add(7)(q21),-15,der(15)t(15;15)(p11. 1;q15,-18[cp5].	*TP53*	10	No				Progressed

40	AML		+ Y, + 21, - 5, -17, -18, and 1 to 4 unidentified "marker" chromosomes	ND	50	Yes	Transfusions	No	Clofarabine+LDAC; 7+3,	Progressed
41	AML	Pure Erythroid Leukemia	43-46,XY,?t(3;19)(q27;p13.3),der(5)t(5;17)(q13;q11.2),der(16;21)(p10;q10),-17, +mar[cp22]/ 46,XY[3].	ND	91	Yes	Transfusions	No	7+3	Progressed
42	MDS		+ 8	ND	0	No	None	No		Stable
43	MDS		45,XX,- 5,-7, +mar[16]/46,XX[4]	ND	0	NA				NA

MDS, myelodysplastic syndrome; AML, acute myeloid leukemia; RAEB-1,refractory anemia with excess blasts-1; RAEB-2, refractory anemia with excess blasts-2; RCMD, reftactory anemia with multilineage dysplasia; MDS/MPN-U, myelosyplastic and myeloproliferative syndromes- unclassified; CK, complex karyotype; MK, monosmal karyotype; ESA, erythropoietin stimulating agents ; G-CSF, granulocyte-colonony stimulating factor

Supplementary Table 2: Characteristics, therapies, and outcomes for patients who underwent stem cell transplant for therapy-related myeloid neoplasm (t-MN)

Morphologic classification	BM blasts (%)	Complex karyotype?	Pre-SCT therapy	Donor	Conditioning	Vital status	Cause of death	Survival from t-MN (months)
RAEB-2	11	No	HMA	Auto	Bu/Cy	Died	Progressive MM	120
Pure erythroid leukemia	18	Yes	7+3 x 2	MRD	Flu/Mel	Alive	N/A	89
RAEB-2	10	Yes	7+3, MEC	MRD	Bu/Flu	Died	Progressive t-MN	27
RCMD	3	Yes	HMA	Auto	Bu/Cy	Died	Progressive t-MN	20
RAEB-2	15	No	HMA	MUD	Flu/Mel	Died	Progressive t-MN	15
RCMD	2	No	HMA	MRD	Flu/Mel	Died	Transplant-related mortality	14
RAEB-1	7	Yes	None	Auto	Mel 140	Died	Progressive MM/t-MN	12
RAEB-1	2	Yes	HMA	Auto	Mel 200	Died	Progressive t-MN	12

BM – bone marrow; SCT – stem cell transplant; t-MN – therapy related myeloid neoplasm; SCT – stem cell transplant; RAEB – refractory anemia with excessive blasts; RCMD – refractory cytopenia with multilineage dysplasia; HMA – hypomethylating agents; 7+3 – anthracycline with cytarabine; MEC – mitoxantrone, etoposide, and cytarabine; auto – autologous; MRD – matched related donor; MUD – matched unrelated donor; Bu – busulfan; Cy – cyclophosphamide; Flu – fludarabine; Mel – melphalan

Supplementary Table 3. Multivariable cox proportional hazard analysis for overall survival from the time of t-MN diagnosis

Variable	Hazard Ratio	95% Confidence Interval	*P*-value
≥2 lines of treatment pre-allogeneic SCT	1.33	0.57-3.10	0.4962
Total lines of alkylator therapy ≥ 1 *vs*. no additional therapy	4.32	1.52-12.22	**0.0058**
BM blasts ≥10% vs < 10%	2.51	1.13-5.66	**0.0265**
Complex karyotype	2.63	1.09-6.35	**0.0305**
SCT –stem cell transplant; BM – bone marrow			
